# Supplementary material for: Small Molecule RBI2 Disrupts Ribosome Biogenesis through Pre-rRNA Depletion
Source: Cancers (Basel). 2023 Jun 23;15(13):3303. doi: 10.3390/cancers15133303 (PMC10340317; doi:10.3390/cancers15133303)
Supplement: Supplementary file 1 [file cancers-15-03303-s001.zip › cancers-2416098-supplementary.pdf]

Supplemental Figures:

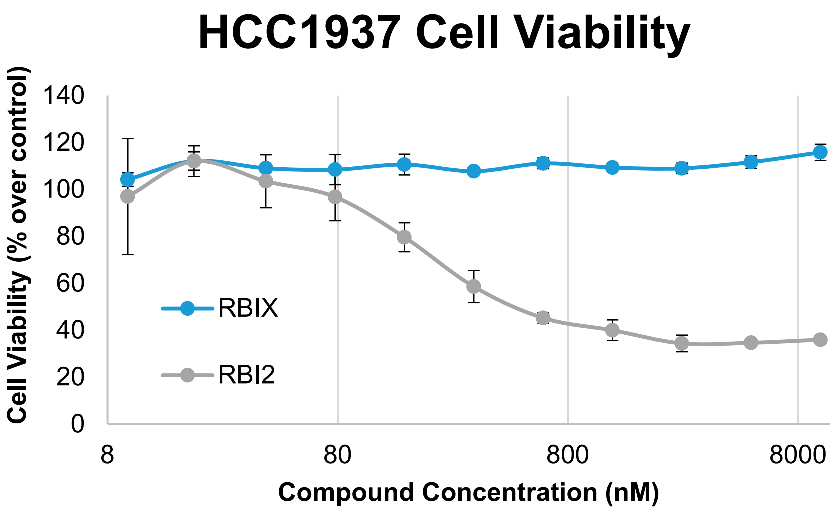

Figure S1: Alamar blue cell viability curve for HCC1937 cell line. Cells were treated with RBI2, or RBIX over a series of concentrations. N=3. Error bars represent standard deviation about the mean.

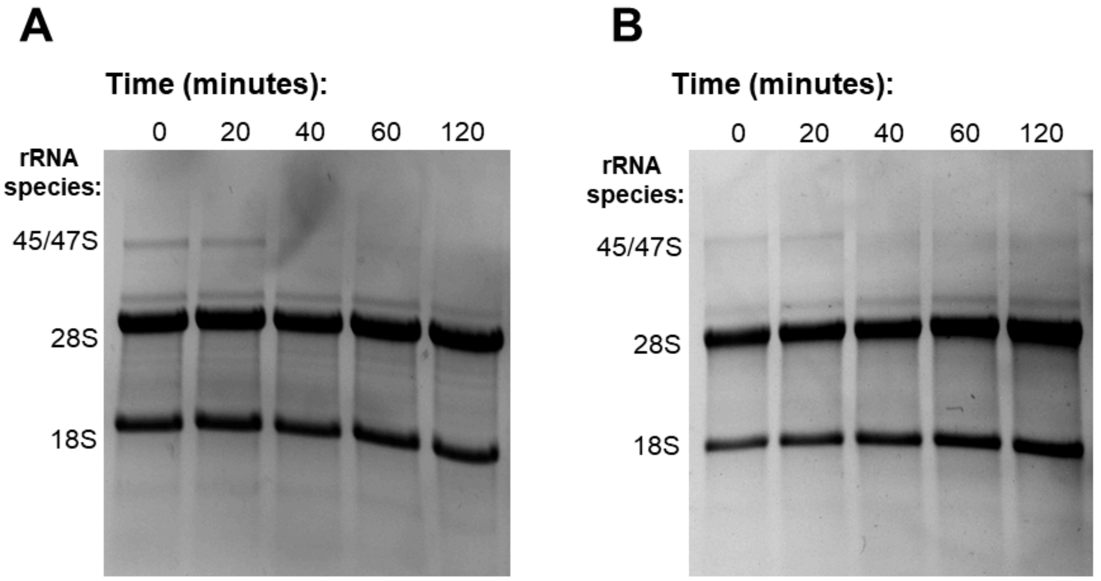

Figure S2: Ethidium bromide staining of RNA isolated from RBI2 treated A) MDA-231 cells and B) HCC-1937 cells displays a decrease in pre-rRNA 45/47S over treatment time, with no observed changes in mature rRNA, 28S and 18S species. Representative gels for n=3.

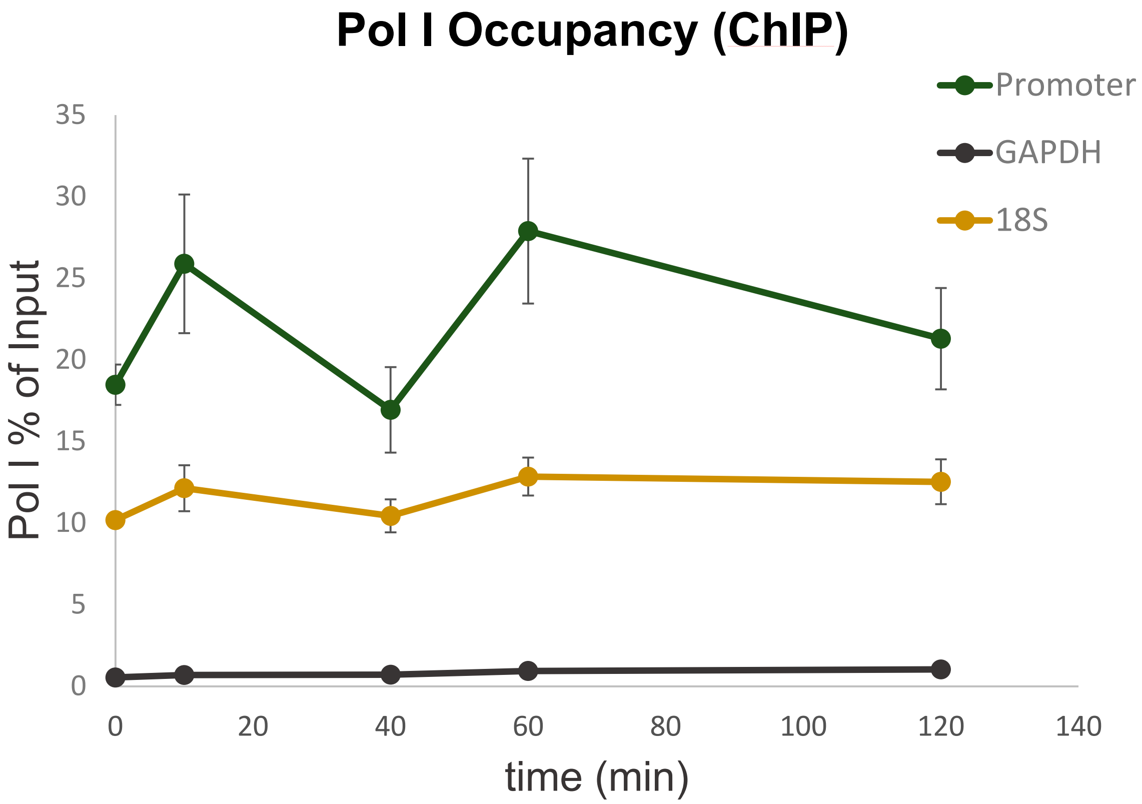

Figure S3: Chromatin immunoprecipitation of Pol I reveals no significant change in Pol I occupancy across the rDNA over time for A375 cells treated with RBI2 at the Promoter, or 18S region of the rDNA. n=3. Error bars represent standard deviation about the mean.

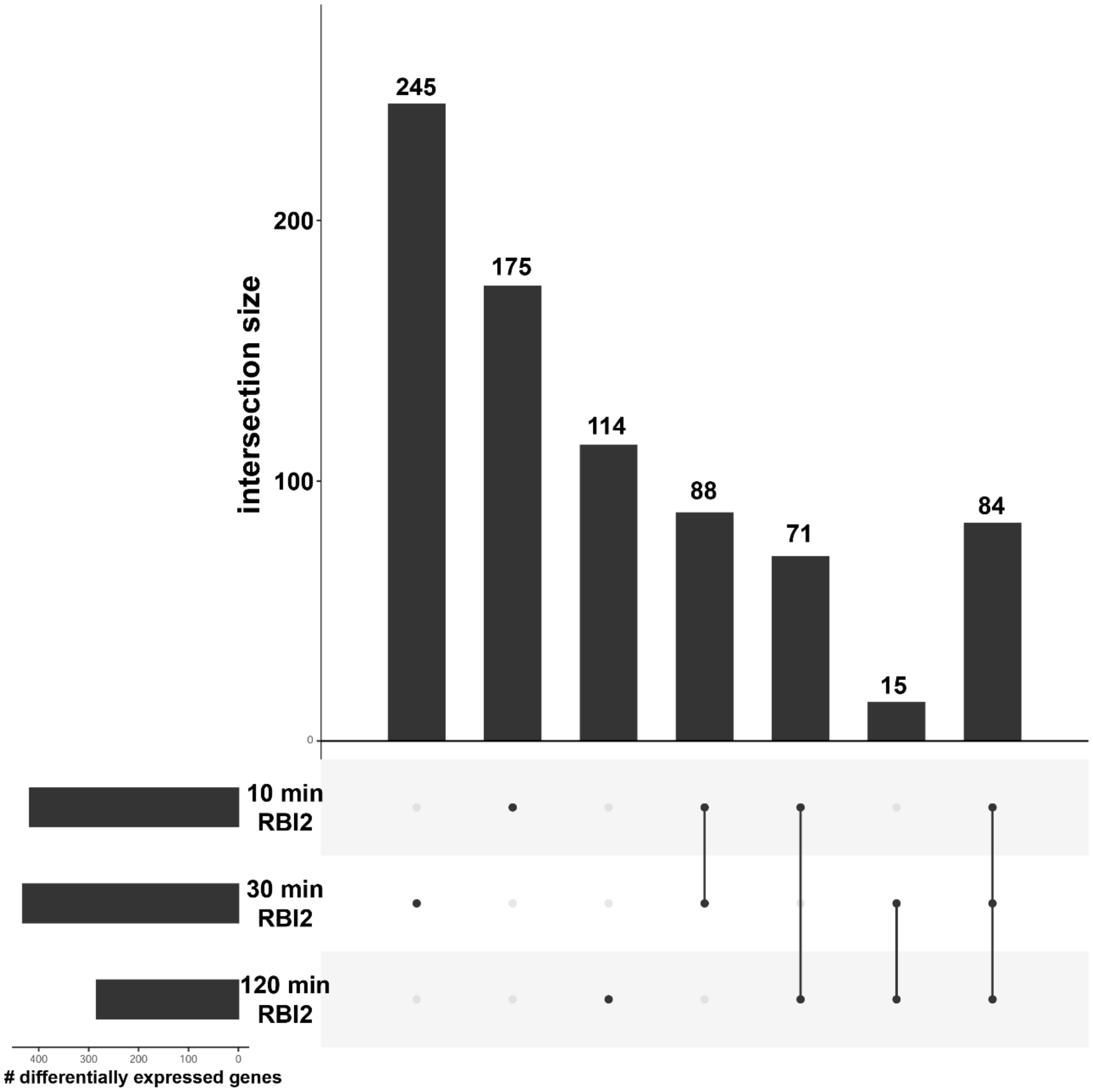

Figure S4: Transcriptional response to RBI2 treatment varies across time points, but a set of subset of genes are consistently differentially expressed (DEGs). Upset plot showing the number of significant (pval < 0.05, absolute log2 fold change > 1) DEGs at each time point, and the number of genes unique to each intersection across time points (Intersection size). 84 Genes were significantly differentially expressed between RBI2 and DMSO treatment conditions with consistent log2 fold change directions.

Gene Ontology Analysis of RBI2 and CX-5461 Enrichment								
	geneSet	description	size	overlap	expect	enrichmentRatio	pValue	FDR
RBI2 over CX-5461 10 minutes	GO:0016569	covalent chromatin modification	468	22	5.1950078	4.23483483	1.20E-08	1.09E-04
	GO:0016570	histone modification	455	21	5.05070203	4.15783784	3.61E-08	1.64E-04
	GO:0051253	negative regulation of RNA metabolic process	1295	37	14.375075	2.57389961	7.81E-08	2.37E-04
	GO:1903507	negative regulation of nucleic acid-templated transcription	1215	35	13.4870395	2.59508397	1.55E-07	2.80E-04
	GO:1902679	negative regulation of RNA biosynthetic process	1217	35	13.5092404	2.59081925	1.61E-07	2.80E-04
	GO:0045892	negative regulation of transcription, DNA-templated	1167	34	12.9542182	2.62462771	1.84E-07	2.80E-04
	GO:0045934	negative regulation of nucleobase-containing compound metabolic process	1418	38	15.7404296	2.41416536	2.62E-07	3.40E-04
	GO:0051276	chromosome organization	1143	33	12.6878075	2.60092218	3.55E-07	3.91E-04
	GO:0009790	embryo development	980	30	10.8784351	2.75774959	3.87E-07	3.91E-04
	GO:2000113	negative regulation of cellular macromolecule biosynthetic process	1359	36	15.0855034	2.38639699	7.62E-07	6.66E-04
	geneSet	description	size	overlap	expect	enrichmentRatio	pValue	FDR
RBI2 over CX-5461 30 minutes	GO:0010629	negative regulation of gene expression	1734	105	50.5654626	2.07651615	1.67E-13	1.10E-09
	GO:2000113	negative regulation of cellular macromolecule biosynthetic process	1359	89	39.6300252	2.24577198	2.41E-13	1.10E-09
	GO:0010558	negative regulation of macromolecule biosynthetic process	1434	91	41.8171127	2.17614259	7.50E-13	1.55E-09
	GO:0051253	negative regulation of RNA metabolic process	1295	85	37.7637105	2.25083814	8.24E-13	1.55E-09
	GO:0009890	negative regulation of biosynthetic process	1516	94	44.2083283	2.12629619	1.11E-12	1.55E-09
	GO:0031327	negative regulation of cellular biosynthetic process	1492	93	43.5084603	2.13751531	1.11E-12	1.55E-09
	GO:0045892	negative regulation of transcription, DNA-templated	1167	79	34.0310812	2.32140729	1.28E-12	1.55E-09
	GO:1903507	negative regulation of nucleic acid-templated transcription	1215	81	35.4308172	2.2861454	1.41E-12	1.55E-09
	GO:1902679	negative regulation of RNA biosynthetic process	1217	81	35.4891396	2.28238839	1.54E-12	1.55E-09

	GO:0045934	negative regulation of nucleobase-containing compound metabolic process	1418	88	41.350534	2.12814664	6.59E-12	5.99E-09
	geneSet	description	size	overlap	expect	enrichmentRatio	pValue	FDR
RBI2 over CX-5461 120 minutes	GO:0010558	negative regulation of macromolecule biosynthetic process	1434	70	23.575903	2.96913335	<10E-16	<10E-16
	GO:2000113	negative regulation of cellular macromolecule biosynthetic process	1359	68	22.3428537	3.04347873	<10E-16	<10E-16
	GO:0031327	negative regulation of cellular biosynthetic process	1492	70	24.5294612	2.85371128	3.33E-16	1.01E-12
	GO:0009890	negative regulation of biosynthetic process	1516	70	24.924037	2.80853379	6.66E-16	1.51E-12
	GO:0010629	negative regulation of gene expression	1734	75	28.5081003	2.63083121	1.67E-15	3.03E-12
	GO:0045934	negative regulation of nucleobase-containing compound metabolic process	1418	65	23.3128525	2.78816159	1.49E-14	2.25E-11
	GO:0051253	negative regulation of RNA metabolic process	1295	59	21.2906516	2.77116929	4.29E-13	5.57E-10
	GO:1903507	negative regulation of nucleic acid-templated transcription	1215	56	19.975399	2.80344838	1.22E-12	1.31E-09
	GO:1902679	negative regulation of RNA biosynthetic process	1217	56	20.0082803	2.79884123	1.30E-12	1.31E-09
	GO:0045892	negative regulation of transcription, DNA-templated	1167	54	19.1862474	2.81451598	2.88E-12	2.61E-09
	geneSet	description	size	overlap	expect	enrichmentRatio	pValue	FDR
CX-5461 over RBI2 10 minutes	GO:1901566	organonitrogen compound biosynthetic process	1776	81	28.1329653	2.879184582	<10E-16	<10E-16
	GO:0043603	cellular amide metabolic process	1033	67	16.3633745	4.094509959	<10E-16	<10E-16
	GO:0033365	protein localization to organelle	888	59	14.0664827	4.194367663	<10E-16	<10E-16
	GO:0006518	peptide metabolic process	780	65	12.3556942	5.260732323	<10E-16	<10E-16
	GO:0043604	amide biosynthetic process	766	64	12.1339254	5.274467917	<10E-16	<10E-16
	GO:0043043	peptide biosynthetic process	636	62	10.074643	6.154064227	<10E-16	<10E-16
	GO:0006412	translation	613	62	9.71030841	6.384967126	<10E-16	<10E-16
	GO:0072594	establishment of protein localization to organelle	537	45	8.50642026	5.290121889	<10E-16	<10E-16
	GO:0022613	ribonucleoprotein complex biogenesis	440	39	6.9698788	5.595506198	<10E-16	<10E-16
	GO:0006605	protein targeting	412	42	6.52634105	6.435458959	<10E-16	<10E-16
	geneSet	description	size	overlap	expect	enrichmentRatio	pValue	FDR
CX-	GO:0033554	cellular response to stress	1867	297	176.662607	1.68117071	<10E-16	<10E-16
	GO:0070727	cellular macromolecule localization	1825	287	172.688408	1.6619529	<10E-16	<10E-16

	GO:0034613	cellular protein localization	1815	286	171.74217	1.66528699	<10E-16	<10E-16
	GO:0007049	cell cycle	1739	292	164.550762	1.7745284	<10E-16	<10E-16
	GO:0022402	cell cycle process	1274	222	120.550702	1.84154879	<10E-16	<10E-16
	GO:0051726	regulation of cell cycle	1106	190	104.653906	1.81550796	<10E-16	<10E-16
	GO:0070647	protein modification by small protein conjugation or removal	1029	196	97.3678747	2.01298427	<10E-16	<10E-16
	GO:0000278	mitotic cell cycle	927	172	87.7162486	1.96086817	<10E-16	<10E-16
	GO:0032446	protein modification by small protein conjugation	790	155	74.7527901	2.0735012	<10E-16	<10E-16
	GO:0016567	protein ubiquitination	738	149	69.8323533	2.1336815	<10E-16	<10E-16
	geneSet	description	size	overlap	expect	enrichmentRatio	pValue	FDR
CX-5461 over RBI2 120 minutes	GO:0051254	positive regulation of RNA metabolic process	1668	46	15.7131885	2.92747713	1.03E-11	9.39E-08
	GO:0045893	positive regulation of transcription, DNA-templated	1499	42	14.1211448	2.9742631	6.66E-11	1.84E-07
	GO:0045935	positive regulation of nucleobase-containing compound metabolic process	1847	47	17.399436	2.70123699	9.35E-11	1.84E-07
	GO:1903508	positive regulation of nucleic acid-templated transcription	1583	43	14.9124565	2.88349542	9.94E-11	1.84E-07
	GO:1902680	positive regulation of RNA biosynthetic process	1584	43	14.9218769	2.88167503	1.01E-10	1.84E-07
	GO:0010628	positive regulation of gene expression	1911	47	18.0023401	2.6107717	2.98E-10	4.51E-07
	GO:1903507	negative regulation of nucleic acid-templated transcription	1215	35	11.4457578	3.0579015	1.87E-09	2.22E-06
	GO:1902679	negative regulation of RNA biosynthetic process	1217	35	11.4645986	3.05287619	1.96E-09	2.22E-06
	GO:0051253	negative regulation of RNA metabolic process	1295	36	12.199388	2.95096771	2.70E-09	2.73E-06
	GO:0010557	positive regulation of macromolecule biosynthetic process	1826	43	17.2016081	2.49976629	8.29E-09	6.75E-06

Figure S5: Top 10 Gene ontology enrichment categories for RBI2 over CX-5461 at 10, 30, and 120 minutes of treatment, and for CX-5461 over RBI2 at 10, 30, and 120 minutes of treatment.

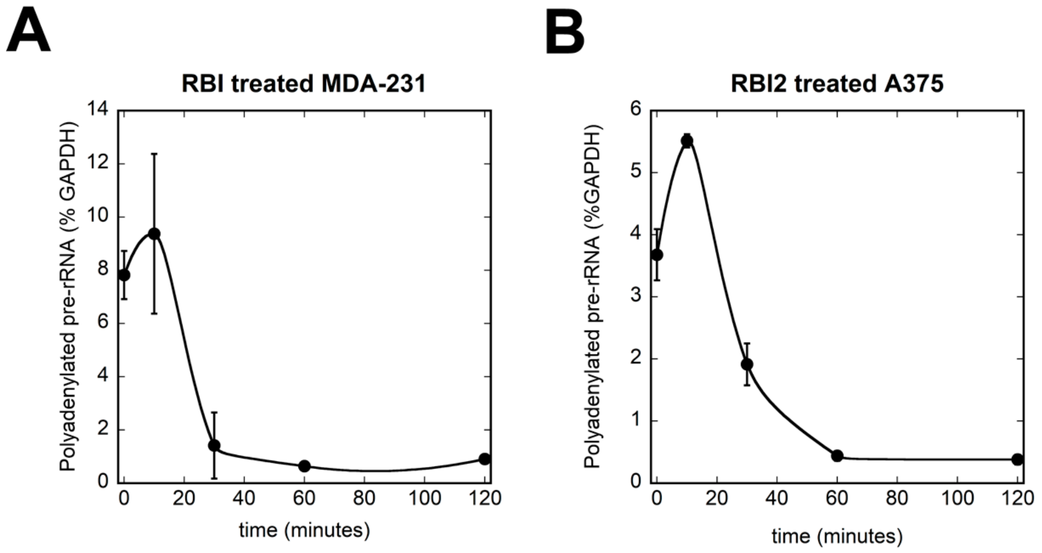

Figure S6: Poly-adenylation of pre-rRNA was measured via RT-qPCR in A) MDA-231 and B) A375 cells. For both cell types, RNA was taken from RBI2 treated cells (the same RNA isolation samples as used in Figure 2) and reverse-transcribed with a Poly-dT oligo. Poly-dT reverse transcribed pre-rRNA cDNA was quantified via qPCR, and normalized to GAPDH RNA abundance. n = 3. Error bars represent standard deviation about the mean.

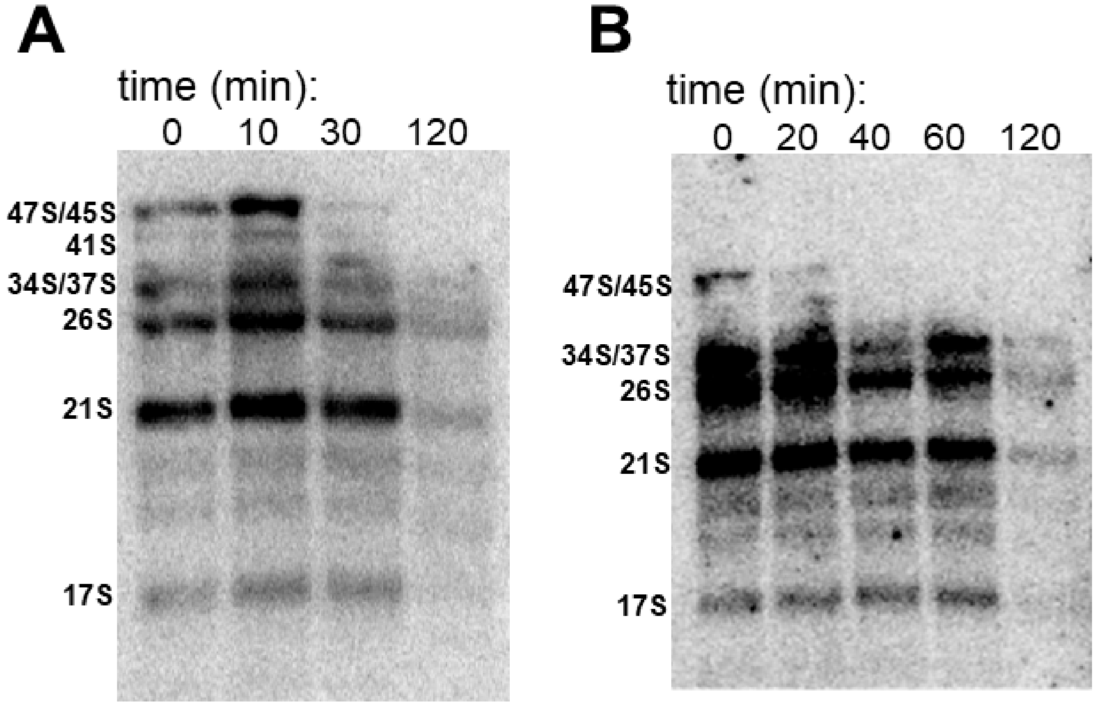

Figure S7: Northern blots for P3 region of RNA isolated from RBI2 treated A) A375 and B) HCC1937 cell lines display a loss of all pre-rRNA species over time, with no appearance of alternative intermediate species. Representative blots for n=3.
